# Complete Chloroplast Genome of *Hypericum perforatum* and Dynamic Evolution in *Hypericum* (Hypericaceae)

**DOI:** 10.3390/ijms242216130

**Published:** 2023-11-09

**Authors:** Xinyu Liu, Yuran Bai, Yachao Wang, Yifeng Chen, Wenpan Dong, Zhixiang Zhang

**Affiliations:** 1 School of Ecology and Nature Conservation, Beijing Forestry University, Beijing 100083, China; liuxinyu@bjfu.edu.cn (X.L.); yrbai@bjfu.edu.cn (Y.B.); yifengchen@bjfu.edu.cn (Y.C.); 2 School of Life Sciences, Fudan University, Shanghai 200437, China; ycwang@fudan.edu.cn

**Keywords:** St. John’s wort, phylogeny, codon usage, substitution rate, intron loss, rearrangement

## Abstract

*Hypericum perforatum* (St. John’s Wort) is a medicinal plant from the Hypericaceae family. Here, we sequenced the whole chloroplast genome of *H. perforatum* and compared the genome variation among five *Hypericum* species to discover dynamic changes and elucidate the mechanisms that lead to genome rearrangements in the *Hypericum* chloroplast genomes. The *H. perforatum* chloroplast genome is 139,725 bp, exhibiting a circular quadripartite structure with two copies of inverted repeats (IRs) separating a large single-copy region and a small single-copy region. The *H. perforatum* chloroplast genome encodes 106 unique genes, including 73 protein-coding genes, 29 tRNAs, and 4 rRNAs. *Hypericum* chloroplast genomes exhibit genome rearrangement and significant variations among species. The genome size variation among the five *Hypericum* species was remarkably associated with the expansion or contraction of IR regions and gene losses. Three genes—*trnK*-UUU, *infA*, and *rps16*—were lost, and three genes—*rps7*, *rpl23*, and *rpl32*—were pseudogenized in *Hypericum*. All the *Hypericum* chloroplast genomes lost the two introns in *clpP*, the intron in *rps12*, and the second intron in *ycf3*. *Hypericum* chloroplast genomes contain many long repeat sequences, suggesting a role in facilitating rearrangements. Most genes, according to molecular evolution assessments, are under purifying selection.

## 1. Introduction

*Hypericum perforatum* L., commonly known as St. John’s Wort, a perennial Hypericaceae herb, is widely distributed in temperate areas of Eurasia and northwestern Africa natively and is an indigenous species of China. *H. perforatum* has been extensively utilized in medicine since the first century AD due to its well-known antidepressant, antibacterial, and anti-inflammatory properties [1]. The species has been naturalized worldwide but has become invasive in Oceania and the American continent. With high levels of genotypic plasticity and variable levels of facultative apomixis, *H. perforatum* has rapidly spread throughout these continents. The species can be divided into four subspecies based on morphology and geographical distribution [2,3].

**Figure 1:**
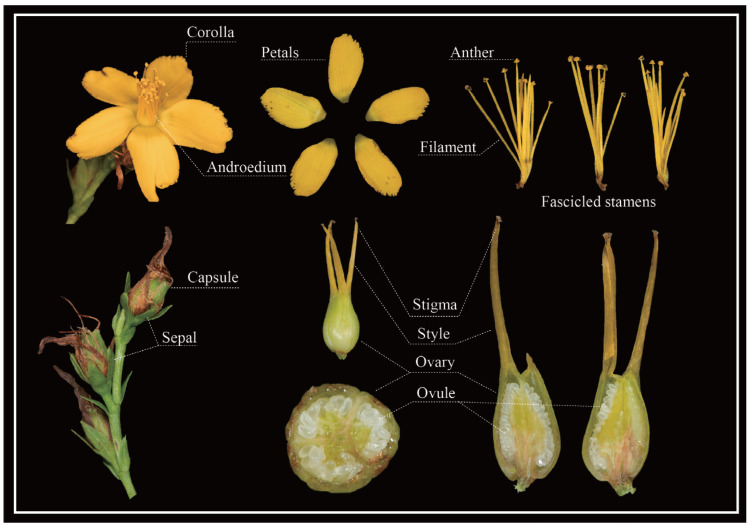
Floral anatomy of *H. perforatum*.

Like other plants in the *Hypericum* genus, *H. perforatum* possesses clustered stamens and glands, which are the shared characteristics of the genus. It distinguishes itself from others by its stamens fascicled into three bundles, translucent glands on its stem and leaves, and dark glands on its flowers (Figure 1). *H. perforatum* displays remarkable variations in its morphology, ploidy, and breeding system, which ranges from sex to apomixis [2,3]. More genetic information is essential to studying this species’ genetics, evolutionary origin, and breeding.

Until now, most research on *H. perforatum* has focused mainly on the properties of its constituent compounds; the genetic background and resources remain scarce. The basic systematical location of this species was revealed by a full Bayesian approach using the internal transcribed spacer and three chloroplast DNA sequence regions [4]. Koch et al. [2] used several chloroplast markers and amplified fragment length polymorphism to present the phylogeographic scenario for the origin of *H. perforatum*; they also discussed the impact of interspecific gene flow. Using the sequence-related amplified polymorphism (SRAP) markers to access the genetic diversity of *H. perforatum* in the Qinling Mountains of China, SRAP markers were shown to be closely associated with morphological variability [5]. However, these molecular markers are less variable and this limits the development and utilization of this medicinal plant.

The chloroplast genome has been widely used for reconstructing relationships and species identification at the species level [6,7,8] or as a molecular marker for genetic diversity analyses within a species [9,10,11]. The typical angiosperm chloroplast genome contains a large single-copy region (LSC), a small single-copy region (SSC), and two inverted repeats (IRs) regions. Although highly conserved in their structural organization, chloroplast genomes may exhibit rearrangements, including gene or intron loss. The *H. ascyron* chloroplast genome, the first completely sequenced one, exhibits dynamic changes in gene and intron content and structure compared to the typical chloroplast genome [12]. However, the mechanisms that lead to rearrangements in *Hypericum* remain poorly known, indicating a need for further analyses to sequence more *Hypericum* chloroplast genomes and compare the dynamic changes among species of the genus.

In this study, we sequenced and assembled the whole chloroplast genome of *H. perforatum* and performed a comparative genome analysis with other *Hypericum* species. We conducted a comprehensive analysis of *H. perforatum*, including annotating functional genes, analyzing codon usage, and identifying sequence repeats to examine the pattern of chloroplast genome organization and infer the phylogenetic relationships among *Hypericum* species.

## 2. Results

### 2.1. Features of the H. perforatum Chloroplast Genome

**Figure 2:**
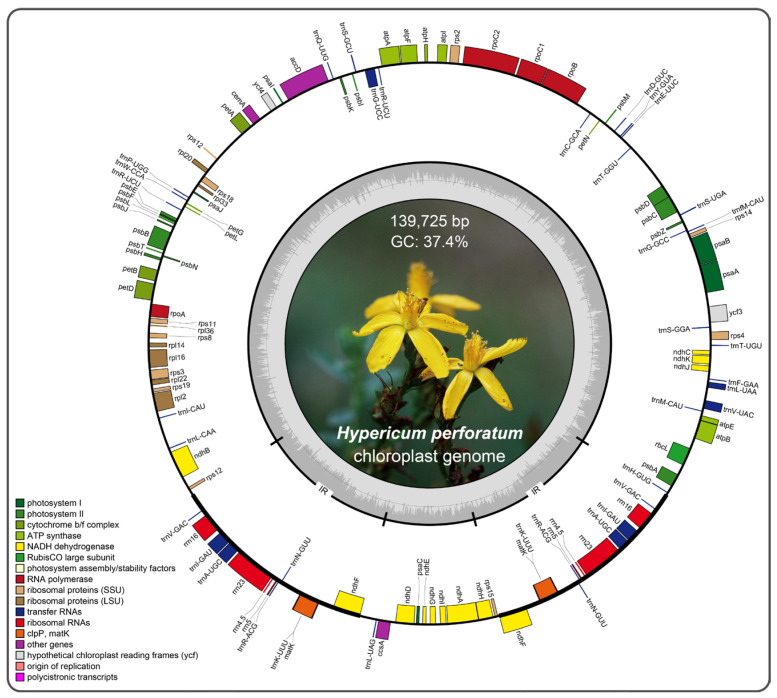
Chloroplast genome map of *H. perforatum*. Genes inside the circle are transcribed clockwise, and those outside are transcribed counterclockwise. Genes within the same functional category are displayed in different colors. The thick lines on the plastid map indicate the inverted repeats (IRs).

The length of the *H. perforatum* chloroplast genome was 139,725 bp, exhibiting a circular quadripartite structure with two copies of IRs of 16,766 bp, separating the LSC region (95,109 bp) and SSC region (11,084 bp) (Figure 2, Table 1). We identified 106 unique genes in the *H. perforatum* chloroplast genome, including 73 protein-coding genes, 29 tRNAs, and four rRNAs (Table S1). Among those genes, 47 were involved in photosynthesis, and 55 were associated with self-replication. Thirteen genes contained an intron; the largest one was found in *ndhA* (1379 bp). The overall GC content was 37.4%.

**Figure 3:**
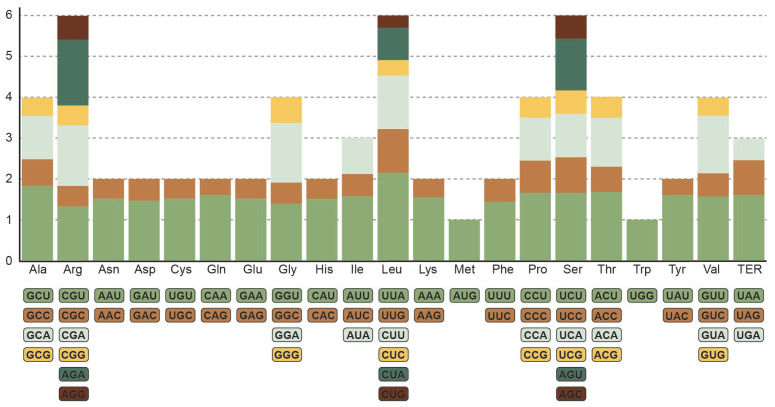
RSCU index for each amino acid and stop codon for the *H. perforatum* chloroplast genome.

The *H. perforatum* chloroplast genome encoded 20,096 codons, representing 20 amino acids and a stop codon (Figure 3). The most abundant amino acid was leucine, encoded by six types of codons, with a frequency of 11.13%, and the second most abundant was isoleucine (1710 codons, 8.5% frequency). Cysteine was the least abundant, with a frequency of 1.16%. Additionally, the most frequent codon was the isoleucine-encoding AUU, with 900 occurrences, while the least frequent was the cysteine-encoding UGC, with 56 occurrences. The stop codons were mostly encoded by UAA (39 of 73). Thirty codons had an RSCU value >1, indicating their usage bias in the *H. perforatum* chloroplast genome, among which 29 ended in A or U (Figure 3). The highest RSCU values were exhibited in leucine-encoding UUA, followed by alanine-encoding GCU and tyrosine-encoding ACU; UGG and AUG exhibited no bias (RSCU = 1).

### 2.2. Comparison of Whole Hypericum Chloroplast Genomes

The chloroplast genomes of the studied *Hypericum* species displayed striking size variations, ranging from 136,817 bp (*H. uralum*) to 162,286 bp (*H. ascyron*) (Table 1). The genome size variation was remarkably associated with the expansion or contraction of IR regions, and IR regions ranged from 3231 bp to 26,846 bp. The GC contents displayed significant differences, ranging from 37.1% to 38.1%. Specifically, the GC content in the IR regions was higher than in the LSC and SSC regions, except for the chloroplast genome of *H. hookerianum*.

Overall, the *Hypericum* chloroplast genomes encoded 103–107 unique genes, including 70–74 protein-coding genes, 29 tRNAs, and four rRNAs (Table 1). Variations were observed when putative pseudogenization or gene losses occurred in some genomes. The *ycf1* and *ycf2* genes were lost in *H. perforatum*, *H. hookerianum*, *H. monogynum*, and *H*. *uralum*. The *rps7*, *rpl23*, and *rpl32* genes were pseudogenized in all *Hypericum* species. Additionally, all five genomes seemed to have lost *trnK-UUU*, *infA*, and *rps16*. The two introns in *clpP,* the intron in *rps12*, and the second intron in *ycf3* were also lost in the five studied genomes.

**Figure 4:**
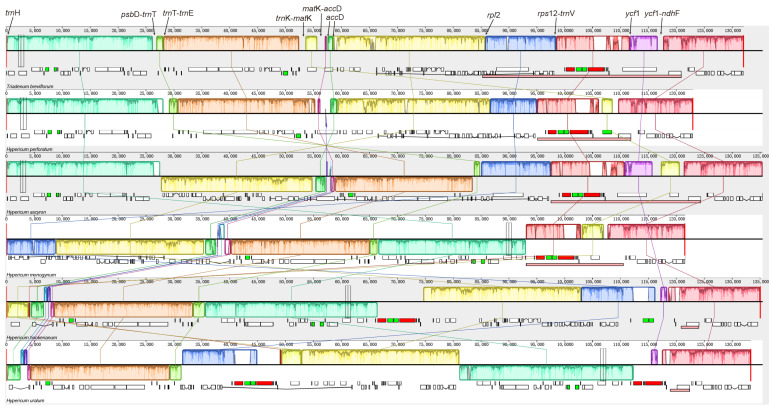
Structural alignments of five *Hypericum* chloroplast genomes using Mauve. The colored blocks represent colinear sequence blocks shared by all chloroplast genomes. Connecting lines indicate the covariate association between blocks. Pink boxes below each plastome block indicate its inverted repeat (IR).

Multiple sequence alignments revealed a highly rearranged structure and large inversions in the five *Hypericum* chloroplast genomes (Figure 4). Ten synteny blocks were identified among the five chloroplast genomes aligned. *H. monogynum* and *H. hookerianum* exhibited a significantly different structure from the other three species. A large inversion (~57 kb) in the LSC region flanking *trnE-UUC* and *rpl2* was found in *H. perforatum* compared with *T. breviflorum*. Several small inversions were variably located in the chloroplast genomes, such as the *trnT-GGU*. The location of *matK* was very different in the five chloroplast genomes; for example, *matK* was located in the LSC region of *T. breviflorum, H. hookerianum*, and *H. monogynum,* while it was transferred from the LSC region into the IR region in *H. perforatum*, *H. ascyron*, and *H. uralum*.

### 2.3. Analysis of Long Sequence Repeats and SSRs

**Figure 5:**
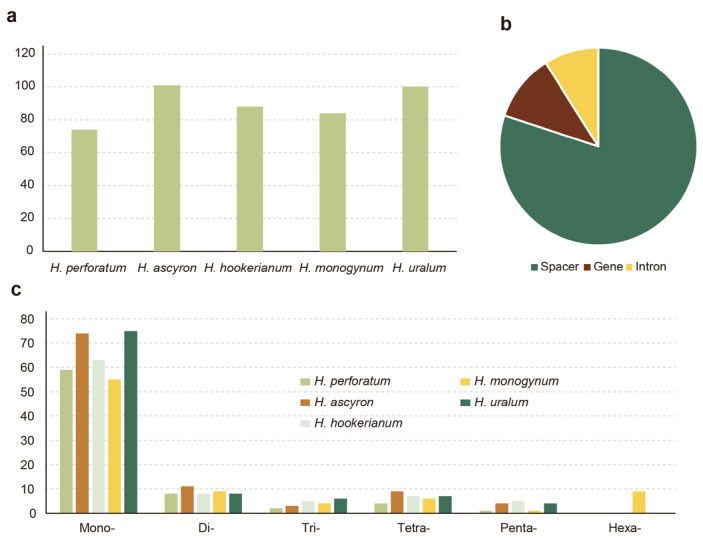
Number and distribution of SSRs. (**a**) Total number of SSRs in each chloroplast genome. (**b**) Distribution of the SSRs in the chloroplast genome. (**c**) Six types of SSR with their length across the chloroplast genome.

The characteristics of SSRs in the five *Hypericum* chloroplast genomes were analyzed, and the pattern of SSR distributions is presented in Figure 5 and Table S2. *H. perforatum* had the lowest number of SSRs (74), while *H. ascyron* had the highest number (101) among the five species. The most abundant SSRs were A or T nucleotide repeats, which accounted for 70.9% of the total (317 of 447). The most abundant type of dinucleotide SSR was AT. The occurrences of mono-, di-, tri-, tetra-, penta-, and hexanucleotides were 72.9%, 9.8%, 4.5%, 7.4%, 3.4%, and 2.0% of the total, respectively. SSRs were mainly distributed in the intergenic spacer region (359 occurrences, 80%), followed by the coding region (49 occurrences, 11%) and introns (40 occurrences, 9%).

**Figure 6:**
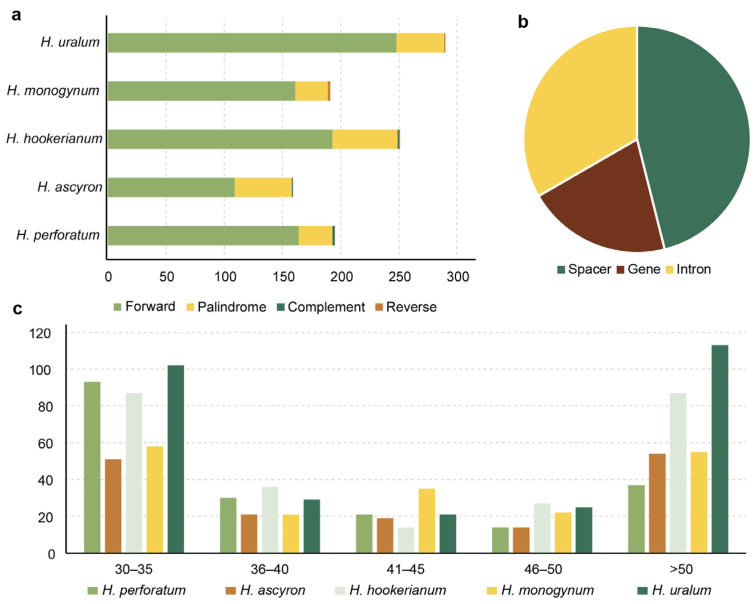
Dispersed repeats in five *Hypericum* chloroplast genomes. (**a**) Types and numbers of repeats. (**b**) Distribution of the repeats. (**c**) Repeat lengths in the five *Hypericum* chloroplast genomes.

The REPuter data were screened and provided information about the four repeat sequences in the five studied chloroplast genomes (Figure 6 and Table S3). In total, 1086 pairs of long repeats were identified in the five genomes, including 875 forward, 203 palindromic, four reverse, and four complementary repeats (Figure 6a). *H. uralum* had the highest numbers, with 248 forward, 41 palindromic, one reverse, and no complementary repeats. *H. ascyron* displayed the lowest numbers with 109 forward, 49 palindromic, one reverse, and no complementary repeats. Most long repeats were distributed in the non-coding regions and a few in coding regions, such as *accD*, *clpP*, and *rps18* (Figure 6b and Table S3). Overall, long repeats varied from 30 bp to 10,138 bp, but most were 30–35 bp and >50 bp (Figure 6c). The longest repeat occurred in the *rps7-trnV* and *trnH-trnV* genes of *H. hookerianum*.

### 2.4. Molecular Evolution of the Hypericum Chloroplast Genome

**Figure 7:**
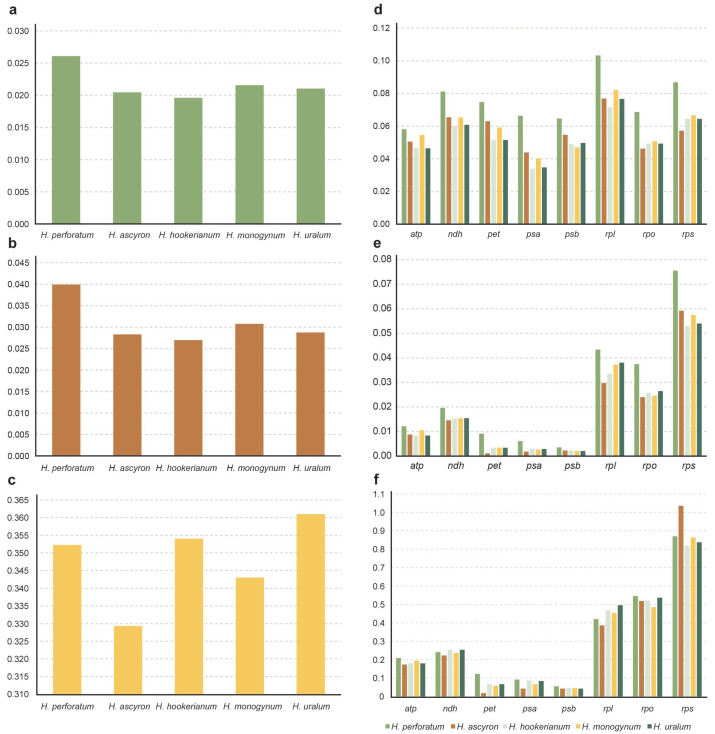
Evolutionary values of *dN*, *dS*, and ω in five *Hypericum* species. (**a**–**c**) *dS*, *dN*, and ω values of the five species. (**d**–**f**) *dS*, *dN*, and ω of different gene groups in the five species.

To decipher the situation of evolutionary rates among *Hypericum* species, we calculated the *dN*, *dS*, and ω values for 63 single protein genes, gene groups, and combinations of all 63 protein-coding genes for each *Hypericum* species, using *T. breviflorum* as a reference (Figure 7 and Table S4). The *t*-test for *dN*, *dS*, and ω values indicated significant differences for each of the considered *Hypericum* species, signifying variable molecular evolution rates among genes.

The mean *dN* value was 0.022, and the highest *dN* value was associated with *rps18* (0.127). Five genes (*petG*, *psbI*, *psbL*, *psbM*, and *psbT*) had non-synonymous substitution sites and displayed a *dN* = 0. The mean *dS* value was 0.065, and the highest *dS* value was associated with *rpl22* (0.154), followed by *ccsA* (0.129) and *ndhH* (0.128). The *psbF* gene had no synonymous substitution sites and displayed a *dS* = 0. Most ω values were <1, indicating that purifying selection acted primarily on most genes. Positive selection occurred in nine genes (*cemA*, *rpl14*, *rpl2*, *rpl33*, *rps14*, *rps18*, *rps19*, *rps2*, and *rps3*), where at least one species pair had a ω > 1. The *cemA* gene had the highest average ω value (2.542).

Among the different gene groups, the *rps* group had the highest *dN* value (0.068), followed by *rpl* (0.039) and *rpo* (0.028), and the photosynthetic genes (*psa* and *psb*) had the lowest *dN* values. The *rpl* gene exhibited the highest value (0.082), and *psa* had the lowest (0.044). The ω values of gene groups were <0.5, except *rpo* and *rps* groups (Figure 7). The ω value was >1 in the *rps* group in *H. ascyron*, indicating the presence of negative selection in this group.

At the species level, *H. perforatum* displayed the highest *dN* value (0.026), indicating a higher evolution rate for this species, while *H. hookerianum* had the lowest *dN* value (0.019). The *dS* values displayed no significant difference among the five species. All ω values were <0.5 (between 0.329–0.361), indicating that the frequency of non-synonymous substitutions was much lower than that of synonymous substitutions and were under purifying selection.

### 2.5. Phylogenetic Inferences in Clusioids Clade of Malpighiales

**Figure 8:**
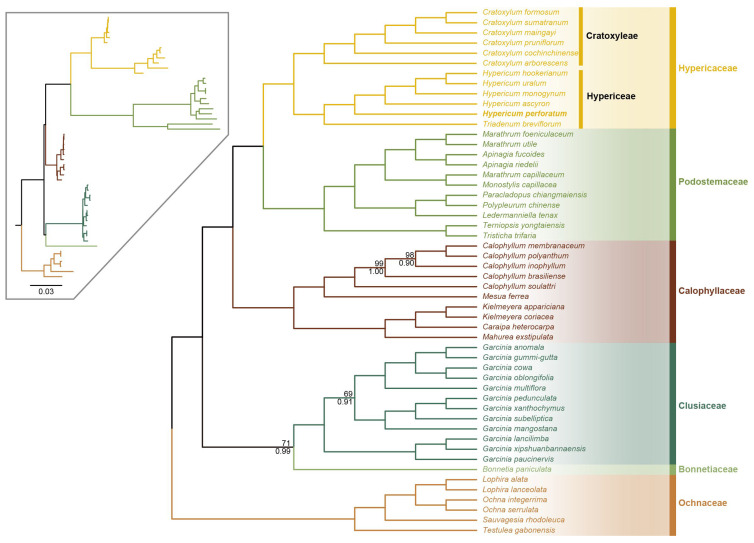
Phylogenetic reconstruction of the Hypericaceae family based on the 63 protein-coding genes. ML bootstrap support value and Bayesian posterior probability are presented at each node. The cladogram on the upper left corner displays the branch length of the phylogenetic tree. Species from the same family are showing in the same color.

To infer the phylogenetic relationships among *Hypericum* species and other genera in clusioids clade of Malpighiales, ML and BI trees were constructed based on 63 coding genes (Figure 8). The topological structures produced by both methods had consistent phylogenies and were highly resolved. The Hypericaceae and Podostemaceae families formed a clade and were related to the Calophyllaceae family with strong support (BS = 100% and PP = 1.00). The Hypericaceae family was split into two clades—Cratoxyleae and Hypericeae—with strong support (BS = 100 and PP = 1.0). The Cratoxyleae tribe included six *Cratoxylum* species, and *C. arborescens* was the first diverged species with high supported values. The Hypericeae tribe included the *Hypericum* and *Triadenum* genera. The five *Hypericum* species formed a clade, and *H. perforatum* was the first diverged species.

## 3. Discussion

### 3.1. Structural Variations in Hypericum Chloroplast Genomes

The study of chloroplast structure, content, and arrangement has increased significantly over the past few decades, particularly with the advent of high-throughput sequencing [6,8,13], which has completely changed many facets of evolutionary biology. For instance, despite numerous findings supporting the overall conserved structure of the chloroplast genome in land plants, a growing body of evidence points to variations among different plant lineages, casting doubt on the idea that plant lineages share an overall conserved structure. Several plant groups, such as Cyperaceae [14], Opuntieae [15], Lobeliaceae [16], and *Medicago* [17], exhibited a high number of structural rearrangements. In this study, we sequenced the chloroplast genome of *H. perforatum* and analyzed the evolution history of the *Hypericum* genus, revealing structural variations (Figure 4). The *Hypericum* chloroplast genome displayed dynamic changes, including inversions, rearrangement, and IR boundary shifts (Figure 4).

Several mechanisms can explain the rise and maintenance of structural variations in the chloroplast genomes. One cause includes inversions and deletions that can result from intra- or intermolecular homologous recombination, leading to structural rearrangements of the chloroplast genome [18,19].

Repeats, especially long ones, which occur very frequently at rearrangement endpoints, were highly linked with rearrangements in the chloroplast genome. Such associations are found in several plant lineages, such as *Jasminum*, *Menodora* [20], *Trifolium* [21], and *Carex* [22]. We identified many long repeats in the *Hypericum* chloroplast genome (Figure 6), suggesting that the repeats might facilitate rearrangements [12].

The integrity of chloroplastic DNA is thought to deteriorate during plant growth due to the degradation of broken but unrepaired molecules, making chloroplast DNA recombination, replication, and repair processes essential for its maintenance [23]. Due to their harsh environment, cacti are exposed to many factors that can cause DNA damage [15,24]. Like cacti, *Hypericum* species usually occupy harsh environments, such as rocky terrain, calcareous areas, dry-to-moist grasslands, acidic fens, or shallow swamps [25], which could explain the significant variation observed in the *Hypericum* chloroplast genomes.

About 20% of angiosperm species have biparental chloroplast inheritance [26,27], and the chloroplast genome rearrangement events are related to this inheritance pattern [7]. *Hypericum* is also a group with biparental chloroplast inheritance [26]. This biparental inheritance might influence the *Hypericum* chloroplast genome rearrangements.

### 3.2. Genome Size Variations and Gene Losses

Typical angiosperm chloroplast genomes range from 130 kb to 170 kb; in related species, the genome size is very similar. However, the chloroplast genome size displayed striking variations among the five *Hypericum* species. The genome size of *T. breviflorum* and *Cratoxylum cochinchinense*, the sister groups of *Hypericum*, were 167,693 bp and 157,103 bp, respectively [28,29]. These results indicate that the smaller genome size of *Hypericum* was contracted during the evolutionary process, according to the phylogenetic relationships of Hypericaceae. IR boundary shifts and gene losses affected the chloroplast genome size. The IR regions of *H. hookerianum* and *H. uralum* were only 3231 bp and 3477 bp, respectively, which could lead to a smaller chloroplast genome. The *ycf2* and *ycf1* genes were the first and second longest genes in the chloroplast genome, and the total length of the two genes amounted to about 12 kb. The two genes were lost in the *H. perforatum* chloroplast genome, which may explain the smaller genome size of this species. In addition, the chloroplast genome size of *H. monogynum* was similar to that of *H. perforatum*. However, the IR length of the *H. monogynum* chloroplast genome was 11 kb, suggesting that the genome size variations in *Hypericum* involve multiple evolutionary mechanisms.

Two coding genes, *infA* and *rps16*, and the tRNA gene *trnK-UUU* have been lost during the evolution of the *Hypericum* chloroplast genome. In angiosperms, the *trnK-UUU* intron includes the *matK* gene and has been found in *Cuscuta* [30], *Isoetes* [31], and *Helicanthes* [32]. The matK gene was retained as a free-standing gene and was transferred to the IR region in the *Hypericum* chloroplast genome. The loss of *trnK-UUU* might be linked to this mutational event. The phylogenetic results showed that the *infA* gene was lost in Malpighiales [12]. Numerous losses of *rps16* were recorded in seed plants [33,34]. Furthermore, three genes (*rps7*, *rpl23*, and *rpl32*) appeared to be pseudogenes due to frameshift indels or internal stop codons in all five *Hypericum* chloroplast genomes. The *rps7*, *rps16*, *rpl23*, and *rpl32* genes encode ribosomal proteins, and ribosomes are necessary for the chloroplast translational apparatus [35]. Many studies have demonstrated that gene losses or pseudogenes in the chloroplast genome are often associated with functional gene transfer to the nuclear genome, leading to substitution by a nuclear-encoded protein [35,36]. For example, the functional transfer of *rpl22* has been observed in Fagaceae and *Passiflora* [37,38,39]; *infA* undergoes multiple independent transfers to the nucleus [40], and *rps16* was discovered in *Populus* and *Medicago* [17,41]. All five genes—*infA*, *rps7*, *rps16*, *rpl23*, and *rpl32*—were found to have functional replacements in the nuclear genome of *H. ascyron* [12]. Further studies are required to evaluate whether these lost genes transfer to the nuclear genome in other *Hypericum* species.

## 4. Materials and Methods

### 4.1. Plant Materials, DNA Extraction, and Sequencing

The material of *H. perforatum* was collected from Yining, Xinjiang, China, and voucher specimens were deposited in the Museum of Beijing Forestry University (BJFC). Identification was conducted by Zhixiang Zhang. The chloroplast genomes of four *Hypericum* species of *H. ascyron* (MZ424306), *H. hookerianum* (MZ714015), *H. monogynum* (ON080517) and *H. uralum* (OP580940) were downloaded from GenBank and added to this study for analysis.

Total DNA was extracted using a modified cetyltrimethylammonium bromide method [42]. The DNA quality was detected by 0.8% agarose gel electrophoresis. The total DNA was fragmented using an ultrasonic homogenizer and used to construct the 350-bp insert library. The library was sequenced using the DNBSEQ-T7 PE150 platform at Novogene Biotechnology Co., Ltd. (Tianjin, China), and approximately 5 GB of data were obtained.

### 4.2. Chloroplast Genome Assembly and Annotation

A quality control of raw data was performed by Trimmomatic 0.36 [43] using the default parameters. The chloroplast genome of *H. perforatum* was assembled using the GetOrganelle toolkit [44] with k-mer lengths of 95, and the genome annotation was performed using the Plastid Genome Annotator (PGA) software (https://github.com/quxiaojian/PGA) [45]. Furthermore, Geneious Prime was used to manually check and revise the annotation errors and missing genes. The annotated chloroplast genome was submitted to GenBank with the accession number OR449303, and the genome map was drawn and visualized using OGDRAW [46]. We calculated codon usage and the relative synonymous codon usage (RSCU) using the MEGA-X program [47] to display the codon usage bias of *H. perforatum* chloroplast genome.

### 4.3. Analysis of Simple Sequence Repeats and Dispersed Repeats

Dispersed repeats and simple sequence repeats (SSRs) were identified in the five *Hypericum* chloroplast genomes using the REPuter online program [48] and Perl script MISA [49], respectively. The REPuter program was employed to identify four types of dispersed repeats, including forward, reverse, palindromic, and complement, in the five *Hypericum* chloroplast genomes. The minimal repeat size was set to 30 bp, and the hamming distance was set to 3 after removing one IR copy from each chloroplast genome. MISA was used to identify the SSRs, with minimal iterations of 10 repeat motifs for mononucleotides, five for dinucleotides, four for trinucleotides, and three for the rest.

### 4.4. Molecular Evolution in Hypericum Chloroplast Genomes

We estimated the non-synonymous substitution (*dN*), synonymous substitution (*dS*), and *dN*/*dS* ratio (ω) to address the evolutionary features among the *Hypericum* chloroplast genomes. This estimation allowed us to elucidate the evolutionary rates and the role of natural selection in molecular evolution. *Triadenum breviflorum* was used as the reference species for these analyses. All the protein-coding genes were extracted according to the genome annotations and aligned using MAFFT. The *dN*, *dS*, and ω values were estimated by YN100 in the PAMLX software [50]. We calculated these values for gene groups with the same function and the species level combined with all coding genes for each species.

### 4.5. Comparison of Hypericum Chloroplast Genomes

We determined synteny and identified possible rearrangements among the five *Hypericum* chloroplast genomes using Mauve v2.4.1 [51] in Geneious Prime with default parameters. The chloroplast genome of *T. breviflorum* was used as a reference. This analysis utilized mauveAligner as the alignment algorithm.

### 4.6. Phylogenetic Analysis

To pinpoint systematic positions of Hypericaceae and reconstruct phylogenetic relationships among the Hypericaceae species, we selected the representative chloroplast genomes of the clusioids clade in the Malpighiales order and all the published chloroplast genome of Hypericaceae from GenBank. Sixty-three protein-coding genes were extracted according to the chloroplast genome annotation. All the genes were aligned using MAFFT with default parameters, and trimAl [52] was used to trim the unreliable alignment regions.

Maximum likelihood (ML) and Bayesian inference (BI) were used to infer the phylogenetic relationships. The ML tree was run using RAxML v8.1.24 [53], and 1000 bootstrap repeats were set to assess node support. The BI tree was inferred using MrBayes 3.2 [54], with a Markov chain Monte Carlo algorithm for 5,000,000 generations, using four simultaneous chains with a random starting tree and default priors. The first 25% of generations were discarded as burn-in, and the remaining trees were determined from the 50% majority-rule consensus to estimate posterior probabilities.

## 5. Conclusions

In this study, the chloroplast genome of *H. perforatum* was sequenced and compared with four other published *Hypericum* species including *H. ascyron*, *H. hookerianum*, *H. monogynum*, and *H. uralum*. Our analysis revealed the *Hypericum* chloroplast genomes exhibit genome rearrangement and significant variations among species. Variation of *Hypericum* chloroplast genomes is remarkable in genome size related to contractions, expansions of IR regions, and the loss of several genes. Three genes were lost and three genes were pseudogenized in *Hypericum.* All the *Hypericum* chloroplast genomes contained a rich number of long repeat sequences, suggesting a role in facilitating genome rearrangements.

## Figures and Tables

**Table 1:** Comparison of the chloroplast genome features of *Hypericum perforatum* and four other *Hypericum* species from Genbank.

Species	*H. perforatum*	*H. ascyron*	*H. hookerianum*	*H. monogynum*	*H. uralum*
Genome size (bp)	139,725	162,286	138,260	138,972	136,817
LSC (bp)	95,109	97,542	120,848	93,123	118,902
SSC (bp)	11,084	11,052	10,950	11,079	10,961
IR (bp)	16,766	26,846	3231	17,385	3477
Overall GC content %	37.4	37.4	38.1	37.8	37.9
GC content in LSC %	35.9	36.5	38.8	36.4	38.6
GC content in SSC %	31.4	32.3	32.6	32.0	32.5
GC content in IR %	43.8	40.0	34.0	43.4	34.9
Unique genes	103	107	105	105	105
Unique protein-coding genes	70	74	72	72	72
Unique rRNAs	4	4	4	4	4
Unique tRNAs	29	29	29	29	29
Pseudogenes	3	3	3	3	3

## Data Availability

The chloroplast genome of *H. monogynum* is deposited in the GenBank database under the following accession number: OR449303.

## Supplementary Materials

The following supporting information can be downloaded at: https://www.mdpi.com/article/10.3390/ijms242216130/s1.
